# Supplementary material for: Insulin/Glucose-Responsive Cells Derived from Induced Pluripotent Stem Cells: Disease Modeling and Treatment of Diabetes
Source: Cells. 2020 Nov 12;9(11):2465. doi: 10.3390/cells9112465 (PMC7696367; doi:10.3390/cells9112465)
Supplement: Supplementary file 1 [file cells-09-02465-s001.pdf]

Insulin/Glucose-Responsive Cells Derived from Induced Pluripotent Stem Cells: Disease Modeling and Treatment of Diabetes

Sevda Gheibi *, Tania Singh, Joao Paulo M.C.M. da Cunha, Malin Fex and Hindrik Mulder *

Unit of Molecular Metabolism, Lund University Diabetes Centre, Jan Waldenströms gata 35; Box 50332, SE-202 13 Malmö, Sweden; tania.singh@med.lu.se (T.S.); joao_paulo.da_cunha@med.lu.se (J.P.M.C.M.d.C.); malin.fex@med.lu.se (M.F.)

* Correspondence: sevda.gheibi@med.lu.se (S.G.); hindrik.mulder@med.lu.se (H.M.)

Supplementary Table 1. Transcription factors associated with development of the pancreas.

Gene	Aliases	Developmental Stage	Function	Ref.
SOX17	SRY-Box Transcription Factor 17	DE, PFE, PSE	Directs the primitive endoderm specification.	[1]
FOXA2	Forkhead Box A2; Hepatocyte Nuclear Factor 3-β	DE, PFE, PSE, PMPs, EPs, Mature β-cells	Establishes lineage-specific transcriptional programs which leads to proper differentiation of stem cells into pancreatic progenitors. Regulates expression of *PDX1* gene and aids in maturation of β-cells	[2]
SHH	Sonic Hedgehog Signaling Molecule	DE	A pleiotropic developmental gene which regulates growth, and differentiation of several organs. Repression of S*HH* expression is vital for pancreas differentiation and development	[3]
CXCR4	C-X-C Motif Chemokine Receptor 4; Stromal Cell-Derived Factor 1 Receptor; Neuropeptide Y3 Receptor	DE	Promotes cell differentiation, proliferation, and survival. Controls the spatiotemporal migration of the angioblasts towards pre-pancreatic endodermal region which aids the induction of *PDX1* expression giving rise to common pancreatic progenitors	[4]
HNF1B	HNF1 Homeobox B Hepatocyte Nuclear Factor 1-β	PFE, PSE, PMPs	Crucial for generation of pancreatic multipotent progenitor cells and $NGN3^+$ endocrine progenitors	[5]
PDX1	Pancreatic and Duodenal Homeobox 1; Insulin Promoter Factor 1; Homeodomain Transcription Factor	PSE, PMPs, EPs, Mature β-cells	Master regulator of pancreatic organogenesis. Induces differentiation from definitive endoderm to pancreatic progenitors. Regulates expression of *Foxa2*, *Gata4*, *Hnf1β*, *Ptf1a*, *Neurog3*, *Nkx6.1*, *Pax6* etc. Activates several key β-cell genes, including *Ins1*, *MafA*, *Glut-2*, *Gck*, and *Iapp*	[6]
GATA4	GATA Binding Protein 4	PSE, PMPs	Expressed in the pancreatic progenitors whereby at later stages of development its expression becomes restricted to mature acinar cells	[7]

SOX9	SRY-Box Transcription Factor 9	PSE, PMPs, EPs	All pancreatic cell lineages are derived from SOX9$^+$. Protects MPCs from apoptosis. Regulates expression of *Hnf1b*, *Hnf6*, *Onecut1* and *Foxa2*. Participates in activating *Ngn3* and *Pdx1* expression	[8]
NEUROG3	Neurogenin 3; NGN3; Class A Basic Helix-Loop-Helix Protein 7; Atoh5; Relax	PMPs, EPs	Activates downstream targets such as *Nkx2.2*, *Nkx6.1*, *Arx*, *Pax4*, *Isl1* and *Neurod1;* is a master switch required for the development of pancreatic endocrine progenitors and successive generation of mono-hormonal endocrine cells	[6,9]
NKX6.1	NK6 Homeobox 1	PMPs, EPs, Mature β-cells	Necessary for generation of mature β-cells and maintaining insulin secretion process. Specific to the pancreas and becomes restricted to β-cells later in the development process	[10–12]
PTF1A	Pancreas Associated Transcription Factor 1a	PMPs	Required for pancreas specification from the foregut endoderm and regulates the expansion of MPCs. Activates exocrine/acinar cell compartment of the pancreas.	[13,14]
GLIS3	GLIS Family Zinc Finger 3	PMPs, EPs, Mature β-cells	Is mainly expressed in preductal and Neurog3$^+$ endocrine progenitors. Regulates expression of *Neurog3*. Participates in insulin gene transcription as well as insulin secretion process	[15,16]
RFX6	Regulatory Factor X6	PMPs, EPs, Mature *α*- and β-cells	Functions in both endoderm development and islet cell differentiation. Directs differentiation of four out of the five islet cells types excluding PP cells. Regulates genes crucial for maturation and function of β-cells	[17–19]
NEUROD1	Neuronal Differentiation 1; Neurogenic Helix-Loop-Helix Protein NEUROD; β-Cell E-Box Transactivator 2	EPs, Mature *α*- and β-cells	Acts as a transactivator of genes crucial for β-cell function and maturation, like insulin gene transcription	[20,21]
NKX2.2	NK2 Homeobox 2	EPs, Mature *α*- and β-cells	Crucial for *α*- and β-cell fate specification and also for maintenance of mature β-cell function by regulating expression of *MAFA* and *GLUT2*	[22,23]
PAX4	Paired Box Protein Pax-4	EPs, Mature β-cells	Required to maintain expression of *PDX1, MNX1*, and *INS* in β-cell precursors. Mis-expression of *PAX4* in *α*-cells transdifferentiates them into β-like cells	[24–26]
PAX6	Paired Box Protein Pax-6	EPs, Mature *α*- and β-cells	Critical for *α*-cell development and regulates transcription of key genes such as *MAFB*, *cMAF* and *NEUROD1*. Regulates glucose-regulated ATP synthesis, Ca2$^+$ dynamics, and β-cell genes	[27–29]

ISL1	ISL LIM Homeobox 1; ISLET1; Insulin Gene Enhancer Protein ISL-1	EPs, Mature α- and β-cells	Crucial for early pancreas morphogenesis, induction of hormone production, survival, and maintenance of physiological responses in endocrine cells. Regulates expression of *Ins*, *Gcg*, *Glut2*, *Pdx1*, *MafA*, and *Arx* etc	[30–32]
ARX	Aristaless Related Homeobox; Homeobox Protein ARX; Cancer/Testis Antigen 121	EPs, Mature α-cells	Crucial for formation of α, PP and β-cells, but later during development it is restricted in specifying α-cell. *Arx* inhibition in mouse α-cells generates β-like cells or its misexpression in β-cells transdifferentiate them into cells exhibiting α or PP-like cells characteristics	[33–35]
RFX3	Regulatory Factor X3	EPs, Mature α- and β-cells	Aids in development and maturation of endocrine cells. Regulates expression of insulin, *Glut2*, and *Gck*.	[36,37]
MAFB	MAF BZIP Transcription Factor B; V-Maf Avian Musculoaponeurotic Fibrosarcoma Oncogene Homolog B	Embryonic and Mature α- & β-cells	Expressed in both embryonic and adult α- and β-cells. Crucial for induction of hormone genes during terminal differentiation and further provides functional maturation to both the cell types	[38–41]
MAFA	MAF BZIP Transcription Factor A; V-Maf Avian Musculoaponeurotic Fibrosarcoma Oncogene Homolog A; RIPE3b1	Mature β-cells	β-cell specific transcription factor. Acts as a transactivator of *Ins* gene transcription in cooperation with Pdx1 and NeuroD1. Regulates genes critical for β-cell function such as *Pdx1*, *Glut2*, *Neurod1*, *Nkx6.1*, *Slc30a8*, and *G6pc2*	[6,40,42–47]
MNX1	Motor Neuron And Pancreas Homeobox 1; HLXB9	Mature β-cells	Crucial for dorsal pancreas specification, β-cell fate determination, maintenance of β-cell identity and inhibition of other endocrine-lineage programs	[48–51]
LDB1	LIM Domain Binding 1	Mature β-cells	Acts as a scaffold to interact with LIM domain factors like ISL1 to form complexes at promotor/enhancer regions to regulate gene expression for successful β-cell terminal differentiation and maturation processes. Regulates expression of *MafA*, *Glp1R*, *Arx*, *Mnx1* and *Glut2*	[32,52,53]

DE, Definitive Endoderm; PFE, Posterior Foregut Endoderm; PSE, Pancreatic-specified endoderm; PMPs, Pancreatic multipotent progenitors; EPs, Endocrine Progenitors.

Reference

1. Yin, C., *Molecular mechanisms of Sox transcription factors during the development of liver, bile duct, and pancreas.* Semin Cell Dev Biol, 2017. **63**: p. 68-78.
2. Gao, N., et al., *Dynamic regulation of Pdx1 enhancers by Foxa1 and Foxa2 is essential for pancreas development.* Genes Dev, 2008. **22**(24): p. 3435-48.
3. Hebrok, M., *Hedgehog signaling in pancreas development.* Mech Dev, 2003. **120**(1): p. 45-57.
4. Katsumoto, K. and S. Kume, *The role of CXCL12-CXCR4 signaling pathway in pancreatic development.* Theranostics, 2013. **3**(1): p. 11-7.

5. De Vas, M.G., et al., *Hnf1b controls pancreas morphogenesis and the generation of Ngn3+ endocrine progenitors.* Development, 2015. **142**(5): p. 871-82.
6. Zhu, Y., et al., *PDX1, Neurogenin-3, and MAFA: critical transcription regulators for beta cell development and regeneration.* Stem Cell Res Ther, 2017. **8**(1): p. 240.
7. Viger, R.S., et al., *Role of the GATA family of transcription factors in endocrine development, function, and disease.* Mol Endocrinol, 2008. **22**(4): p. 781-98.
8. Dubois, C.L., et al., *Sox9-haploinsufficiency causes glucose intolerance in mice.* PLoS One, 2011. **6**(8): p. e23131.
9. Qin, Y., et al., *Pdxl and its role in activating Ngn3 and Pax6 to induce differentiation of iPSCs into islet β cells.* Genet Mol Res, 2015. **14**(3): p. 8892-900.
10. Nelson, S.B., A.E. Schaffer, and M. Sander, *The transcription factors Nkx6.1 and Nkx6.2 possess equivalent activities in promoting beta-cell fate specification in Pdx1⁺ pancreatic progenitor cells.* 2007. **134**(13): p. 2491-2500.
11. Sander, M., et al., *Homeobox gene Nkx6.1 lies downstream of Nkx2.2 in the major pathway of beta-cell formation in the pancreas.* 2000. **127**(24): p. 5533-5540.
12. Taylor, B.L., F.F. Liu, and M. Sander, *Nkx6.1 is essential for maintaining the functional state of pancreatic beta cells.* Cell Rep, 2013. **4**(6): p. 1262-75.
13. Dong, P.D., et al., *Graded levels of Ptf1a differentially regulate endocrine and exocrine fates in the developing pancreas.* Genes Dev, 2008. **22**(11): p. 1445-50.
14. Jin, K. and M. Xiang, *Transcription factor Ptf1a in development, diseases and reprogramming.* Cellular and Molecular Life Sciences, 2019. **76**(5): p. 921-940.
15. Kang, H.S., et al., *Transcription factor Glis3, a novel critical player in the regulation of pancreatic beta-cell development and insulin gene expression.* Mol Cell Biol, 2009. **29**(24): p. 6366-79.
16. Kim, Y.S., et al., *Glis3 regulates neurogenin 3 expression in pancreatic β-cells and interacts with its activator, Hnf6.* Mol Cells, 2012. **34**(2): p. 193-200.
17. Cheng, C., et al., *Identification of Rfx6 target genes involved in pancreas development and insulin translation by ChIP-seq.* Biochem Biophys Res Commun, 2019. **508**(2): p. 556-562.
18. Soyer, J., et al., *Rfx6 is an Ngn3-dependent winged helix transcription factor required for pancreatic islet cell development.* Development, 2010. **137**(2): p. 203-12.
19. Taleb, N. and C. Polychronakos, *RFX6 is needed for the development and maintenance of the β-cell phenotype.* Islets, 2011. **3**(5): p. 291-3.
20. Gu, C., et al., *Pancreatic beta cells require NeuroD to achieve and maintain functional maturity.* Cell Metab, 2010. **11**(4): p. 298-310.
21. Romer, A.I., et al., *Murine Perinatal β-Cell Proliferation and the Differentiation of Human Stem Cell–Derived Insulin-Expressing Cells Require NEUROD1.* 2019. **68**(12): p. 2259-2271.
22. Churchill, A.J., et al., *Genetic evidence that Nkx2.2 acts primarily downstream of Neurog3 in pancreatic endocrine lineage development.* Elife, 2017. **6**.
23. Doyle, M.J. and L. Sussel, *Nkx2.2 regulates beta-cell function in the mature islet.* Diabetes, 2007. **56**(8): p. 1999-2007.
24. Liew, C.G., et al., *PAX4 enhances beta-cell differentiation of human embryonic stem cells.* PLoS One, 2008. **3**(3): p. e1783.
25. Napolitano, T., et al., *Pax4 acts as a key player in pancreas development and plasticity.* Semin Cell Dev Biol, 2015. **44**: p. 107-14.
26. Wang, J., et al., *The concerted activities of Pax4 and Nkx2.2 are essential to initiate pancreatic beta-cell differentiation.* Dev Biol, 2004. **266**(1): p. 178-89.
27. Gosmain, Y., et al., *Glucagon gene expression in the endocrine pancreas: the role of the transcription factor Pax6 in α-cell differentiation, glucagon biosynthesis and secretion.* Diabetes Obes Metab, 2011. **13 Suppl 1**: p. 31-8.
28. Mitchell, R.K., et al., *The transcription factor Pax6 is required for pancreatic β cell identity, glucose-regulated ATP synthesis, and Ca(2+) dynamics in adult mice.* J Biol Chem, 2017. **292**(21): p. 8892-8906.
29. Swisa, A., et al., *PAX6 maintains β cell identity by repressing genes of alternative islet cell types.* J Clin Invest, 2017. **127**(1): p. 230-243.
30. Ahlgren, U., et al., *Independent requirement for ISL1 in formation of pancreatic mesenchyme and islet cells.* Nature, 1997. **385**(6613): p. 257-60.
31. Guo, T., et al., *ISL1 promotes pancreatic islet cell proliferation.* PLoS One, 2011. **6**(8): p. e22387.

32. Wade, A.K., et al., *LIM-domain transcription complexes interact with ring-finger ubiquitin ligases and thereby impact islet β-cell function.* J Biol Chem, 2019. **294**(31): p. 11728-11740.
33. Collombat, P., et al., *Embryonic endocrine pancreas and mature beta cells acquire alpha and PP cell phenotypes upon Arx misexpression.* J Clin Invest, 2007. **117**(4): p. 961-70.
34. Courtney, M., et al., *The inactivation of Arx in pancreatic α-cells triggers their neogenesis and conversion into functional β-like cells.* PLoS Genet, 2013. **9**(10): p. e1003934.
35. Gage, B.K., et al., *The Role of ARX in Human Pancreatic Endocrine Specification.* PLoS One, 2015. **10**(12): p. e0144100.
36. Ait-Lounis, A., et al., *Novel function of the ciliogenic transcription factor RFX3 in development of the endocrine pancreas.* Diabetes, 2007. **56**(4): p. 950-9.
37. Ait-Lounis, A., et al., *The transcription factor Rfx3 regulates beta-cell differentiation, function, and glucokinase expression.* Diabetes, 2010. **59**(7): p. 1674-85.
38. Guo, S., et al., *Inactivation of specific β cell transcription factors in type 2 diabetes.* J Clin Invest, 2013. **123**(8): p. 3305-16.
39. Dai, C., et al., *Islet-enriched gene expression and glucose-induced insulin secretion in human and mouse islets.* Diabetologia, 2012. **55**(3): p. 707-18.
40. Hang, Y. and R. Stein, *MafA and MafB activity in pancreatic β cells.* Trends Endocrinol Metab, 2011. **22**(9): p. 364-73.
41. Cyphert, H.A., et al., *Examining How the MAFB Transcription Factor Affects Islet β-Cell Function Postnatally.* Diabetes, 2019. **68**(2): p. 337-348.
42. Vanderford, N.L., *Regulation of β-cell-specific and glucose-dependent MafA expression.* Islets, 2011. **3**(1): p. 35-7.
43. Aguayo-Mazzucato, C., et al., *MAFA and T3 Drive Maturation of Both Fetal Human Islets and Insulin-Producing Cells Differentiated From hESC.* J Clin Endocrinol Metab, 2015. **100**(10): p. 3651-9.
44. El Khattabi, I. and A. Sharma, *Proper activation of MafA is required for optimal differentiation and maturation of pancreatic β-cells.* Best Pract Res Clin Endocrinol Metab, 2015. **29**(6): p. 821-31.
45. Nishimura, W., S. Takahashi, and K. Yasuda, *MafA is critical for maintenance of the mature beta cell phenotype in mice.* Diabetologia, 2015. **58**(3): p. 566-74.
46. Matsuoka, T.A., et al., *Mafa Enables Pdx1 to Effectively Convert Pancreatic Islet Progenitors and Committed Islet α-Cells Into β-Cells In Vivo.* Diabetes, 2017. **66**(5): p. 1293-1300.
47. Dayer, D., et al., *MafA Overexpression: A New Efficient Protocol for In Vitro Differentiation of Adipose-Derived Mesenchymal Stem Cells into Functional Insulin-Producing Cells.* Cell J, 2019. **21**(2): p. 169-178.
48. Pan, F.C., et al., *Inactivating the permanent neonatal diabetes gene Mnx1 switches insulin-producing β-cells to a δ-like fate and reveals a facultative proliferative capacity in aged β-cells.* Development, 2015. **142**(21): p. 3637-48.
49. Dalgin, G., et al., *Zebrafish mnx1 controls cell fate choice in the developing endocrine pancreas.* Development, 2011. **138**(21): p. 4597-608.
50. Harrison, K.A., et al., *Pancreas dorsal lobe agenesis and abnormal islets of Langerhans in Hlxb9-deficient mice.* Nat Genet, 1999. **23**(1): p. 71-5.
51. Li, H., et al., *Selective agenesis of the dorsal pancreas in mice lacking homeobox gene Hlxb9.* Nat Genet, 1999. **23**(1): p. 67-70.
52. Ediger, B.N., et al., *LIM domain-binding 1 maintains the terminally differentiated state of pancreatic β cells.* J Clin Invest, 2017. **127**(1): p. 215-229.
53. Hunter, C.S., et al., *Islet α-, β-, and δ-cell development is controlled by the Ldb1 coregulator, acting primarily with the islet-1 transcription factor.* Diabetes, 2013. **62**(3): p. 875-86.
